# How avian influenza viruses spill over to mammals

**DOI:** 10.7554/eLife.86051

**Published:** 2023-04-11

**Authors:** Arturo Barbachano-Guerrero, Daniel R Perez, Sara L Sawyer

**Affiliations:** 1 University of Colorado Boulder Boulder United States; 2 University of Georgia Athens United States

**Keywords:** canine H3N2 Influenza virus, airborne transmissibility, HA, PB1, public health, Viruses

## Abstract

The H3N2 canine influenza virus – which originally came from birds – is evolving to become more transmissible between dogs.

**Related research article** Chen M, Lyu Y, Wu F, Zhang Y, Li H, Wang R, Liu Y, Yang X, Zhou L, Zhang M, Tong Q, Sun H, Pu J, Liu J, Sun Y. 2023. Increased public health threat of avian-origin H3N2 influenza virus caused by its evolution in dogs. *eLife* **12**:e83470. doi: 10.7554/eLife.83470.

Picture your local lake covered with migrating geese, ducks or other waterfowl. Even though you don’t hear any coughing, you might well be witnessing ‘flu season’ for birds. Influenza viruses cause gastrointestinal infections in birds, and are spread when birds defecate in water that other birds then drink (Caliendo et al., 2020). Sometimes, however, avian influenza viruses make their way into mammals, including humans, and cause respiratory infections: how does this happen?

Waterfowl are the main natural reservoir for influenza viruses, and influenza viruses that infect humans and other mammals originally came from birds. This spillover can happen in two ways. The first way involves special mammalian hosts (like pigs) that can be infected by both avian and mammalian influenza viruses (Figure 1A). Occasionally, an individual from one of these species becomes simultaneously infected with both types of virus, and the two viruses exchange gene segments to form a novel virus that retains the ability to infect mammals. This process – which is known as gene reassortment – is what happened to start human influenza pandemics in 1957 and 1968 (Harrington et al., 2021).

**Figure 1.**
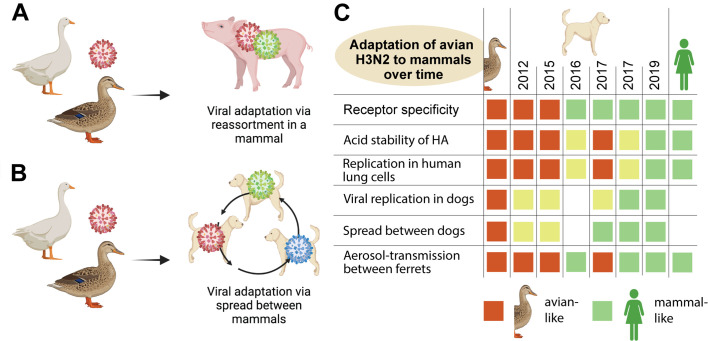
How avian influenza viruses adapt to mammals. (**A**) One way that avian viruses can adapt to mammals is through virus reassortment, as shown here. The avian virus (red) reassorts with another influenza virus that is already adapted to mammals (green), yielding a new mammalian-adapted virus. For this to happen, a single individual must be infected by both viruses simultaneously. (**B**) The other way that avian viruses can adapt to mammals is by direct infection and subsequent adaptation, as is illustrated here. As an avian virus (red) starts to spread between individuals in a mammalian population, it can acquire mutations that make it better at infecting mammals (an evolutionary intermediate is shown in blue). Eventually it may become well-adapted to infecting mammals (green). (**C**) Chen at al. isolated H3N2 canine influenza viruses from samples collected between 2012 and 2019 (middle columns), and tested them for a range of phenotypic properties that are associated with influenza viruses being able to infect mammals (rows). As controls, they included avian H3N2 (left column) and human H3N2 (right column) viruses. They found that, over time, the canine H3N2 virus gained multiple properties that make it compatible with mammals (green squares), with several of the key adaptations occurring around or after 2016/2017. Yellow squares indicate intermediate phenotypes. A blank square indicates that the test was not performed.

The second way that spillover can happen involves a mammal getting directly infected with a bird virus (Figure 1B). This individual then transmits this bird virus to others in its same species. If infection of the new species is sustained over time and within many individuals, the avian virus will experience natural selection for genetic mutations that make it more and more compatible with the mammalian species (Lipsitch et al., 2016).

Now, in eLife, Yipeng Sun (Chinese Agricultural University) and co-workers – including Mingyue Chen and Yanli Lyu as joint first authors – report the results of experiments which shed light on how a virus that normally infects birds is spreading and evolving in dogs around the world via this second form of spillover (Chen et al., 2023). It is important to understand how this happens, because there are avian viruses currently adapting to several different mammalian species via this direct bird-to-mammal pathway (Meng et al., 2022). Each of these ongoing evolution experiments could, at least in theory, yield a human-adapted virus.

There are only a small number of influenza viruses that humans live with, and these constitute our seasonal influenza virus repertoire. The same is true for dogs, and there are just two influenza viruses that dogs transmit consistently between each other – H3N2 and H3N8. The H3N2 canine influenza virus was first found in 2006 in Guangdong Province in China, and research revealed that its genome was closely related to that of the H3N2 influenza viruses found in birds (Li et al., 2010). The virus has since spread out of Asia and was first identified in dogs in the United States in 2015.

To study the evolution of H3N2 in dogs, Chen et al. collected 4174 tracheal swab samples from sick dogs in veterinary hospitals and kennels. The oldest sample dated back to 2012 and the most recent was from 2019. In addition to sequencing the viruses in these samples and analyzing them phylogenetically, the researchers also tested the viruses from a subset of the samples for a range of phenotypic properties that are associated with bird influenza viruses being able to infect mammals (Lipsitch et al., 2016). They found that the H3N2 canine influenza viruses have gained a number of these phenotypic properties since 2012, making them more and more adapted to mammals as time goes by (Figure 1C). To draw these conclusions, Chen et al. performed infections in dogs and ferrets, and also examined the ability of these viruses to infect human cells in tissue culture.

The researchers were also able to map some of the adaptions to specific sets of genetic changes in the virus genome. Some of these adaptations of avian H3N2 to dogs had been observed before, but prior studies focused on strains from 2017 or earlier (Martinez-Sobrido et al., 2020; Tangwangvivat et al., 2022). The remarkable experimental virology demonstrated in these studies is laborious, but critical for assessing the danger that animal viruses pose to humans (Warren and Sawyer, 2023).

Just because the H3N2 canine influenza virus is perfecting itself for dogs does not mean that it will infect humans. Once an avian influenza virus has perfected itself for a particular mammalian species, such as dogs, we don’t know the details of what then dictates its transmission to a different mammalian species, such as humans (Warren and Sawyer, 2019). Thankfully, there have been no reports to date of humans being infected with canine influenza virus.
